# Supplementary figures and images for: Population variability in the generation and selection of T-cell repertoires
Source: PLoS Comput Biol. 2020 Dec 9;16(12):e1008394. doi: 10.1371/journal.pcbi.1008394 (PMC7725366; doi:10.1371/journal.pcbi.1008394)

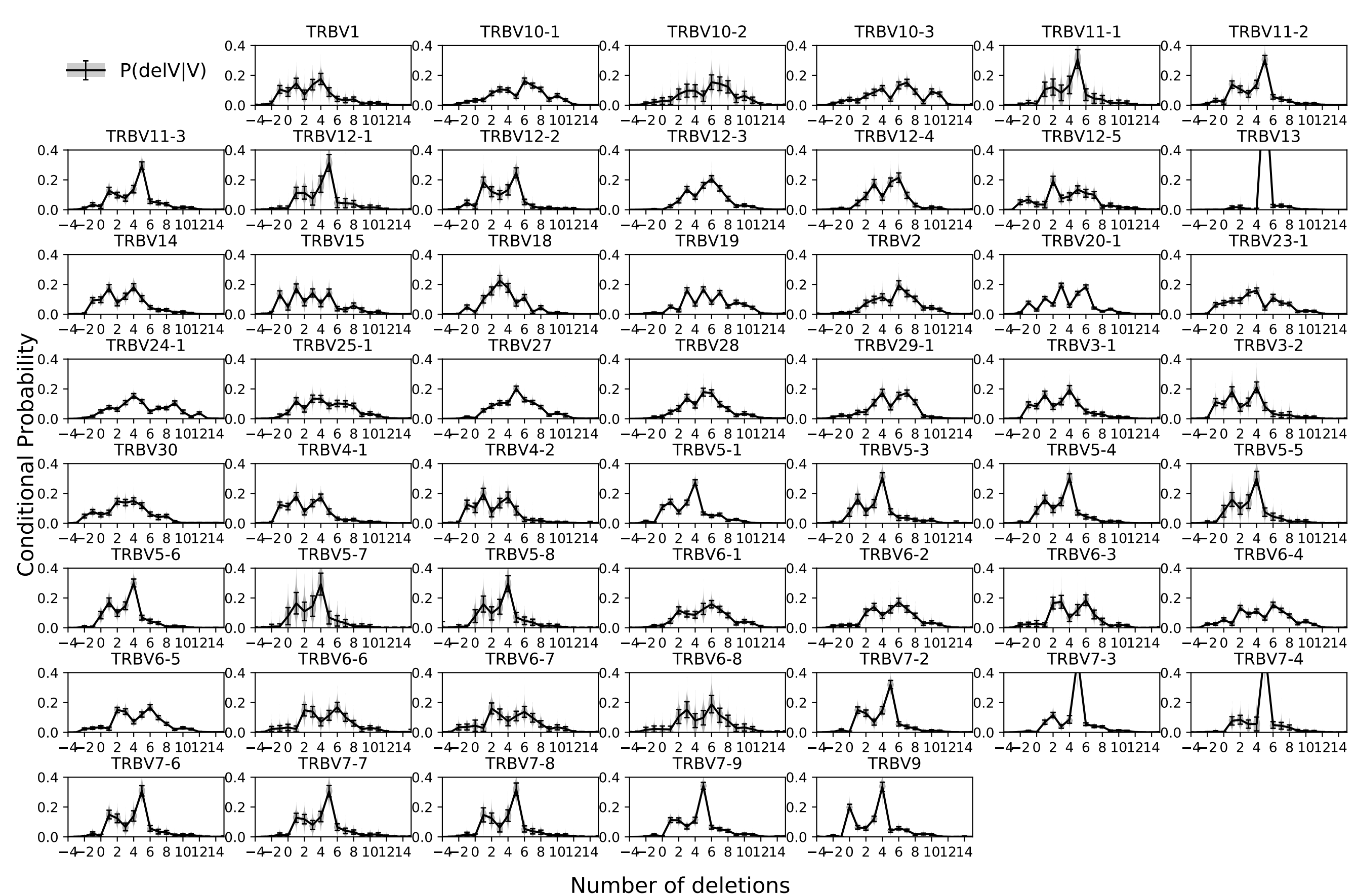

Supplement: S1 Fig — (PDF) [file pcbi.1008394.s001.pdf]

**A**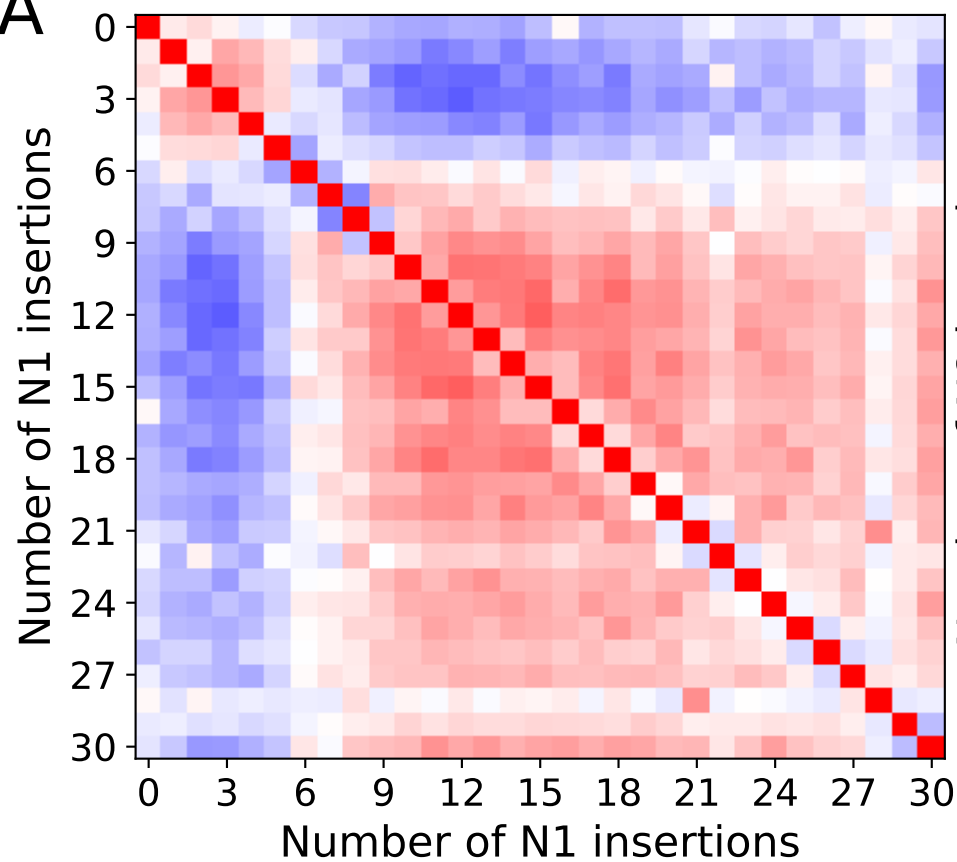**B**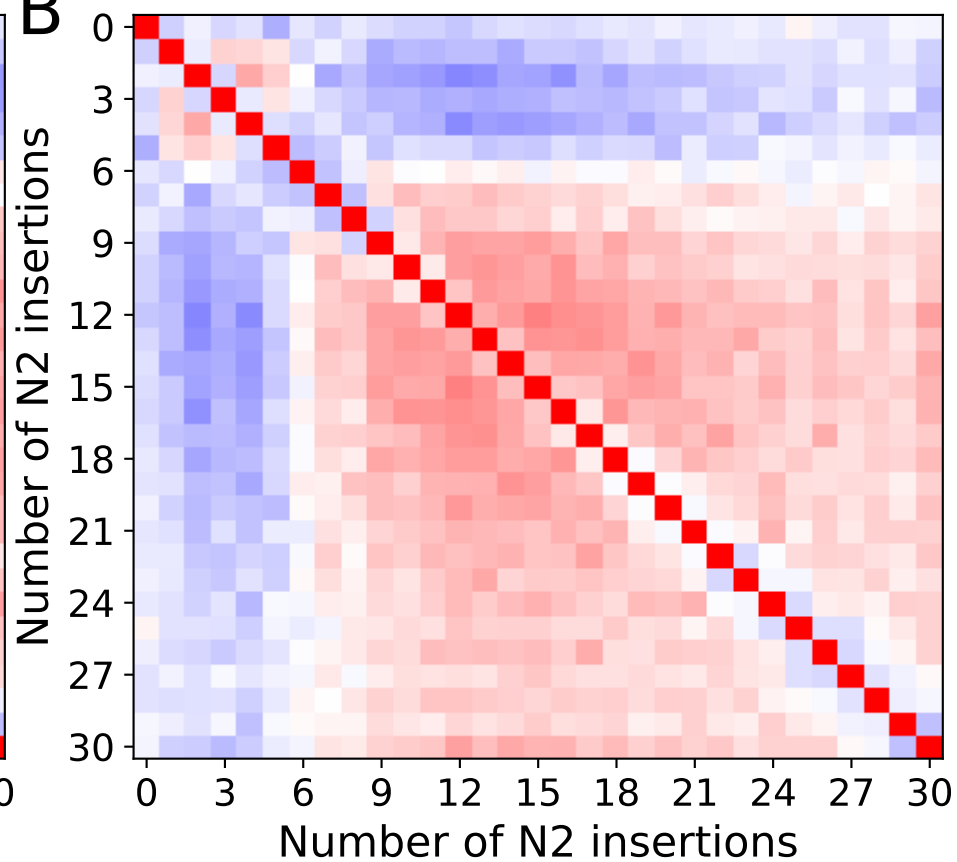**C**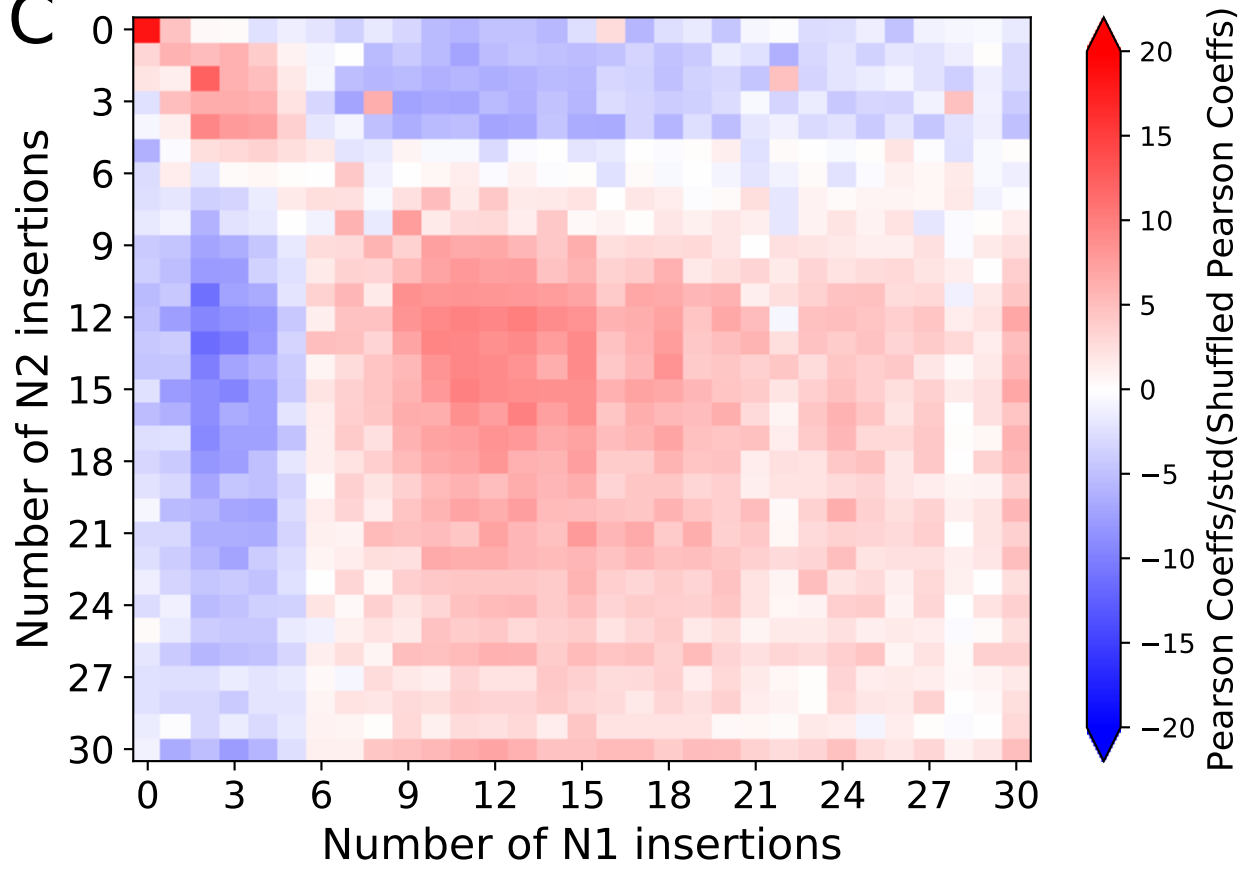

Supplement: S2 Fig — (A) N1-N1 correlations. (B) N2-N2 correlations. (C) N1-N2 correlations. The N1 and N2 distributions are highly correlated over the 651 individual cohort. Rescaling is done by normalizing by the standard deviation of correlation coefficients obtained by shuffling individuals for the two features independently. (PDF) [file pcbi.1008394.s002.pdf]

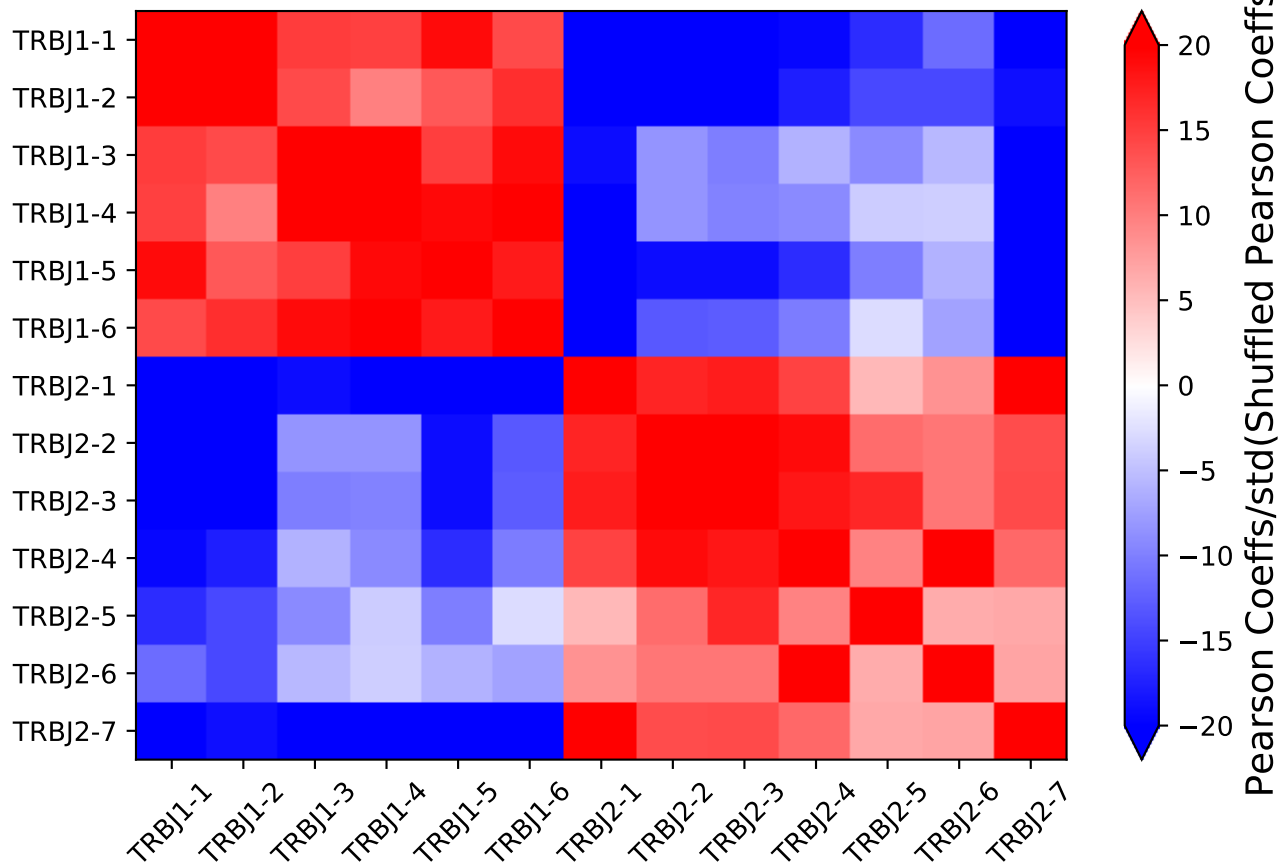

Supplement: S3 Fig — The dominant signal comes from correlations derived from the arrangement of the D and J genes on the chromosome. As genes of the J1 family cannot recombine with the D2 gene, variations in the D usages result in an overall shift in the J1 and J2 gene family usages. This accounts for the strong positive correlation within each J gene family and strong negative correlation between the J1 and J2 families. Rescaling as in S1 Fig. (PDF) [file pcbi.1008394.s003.pdf]

**A**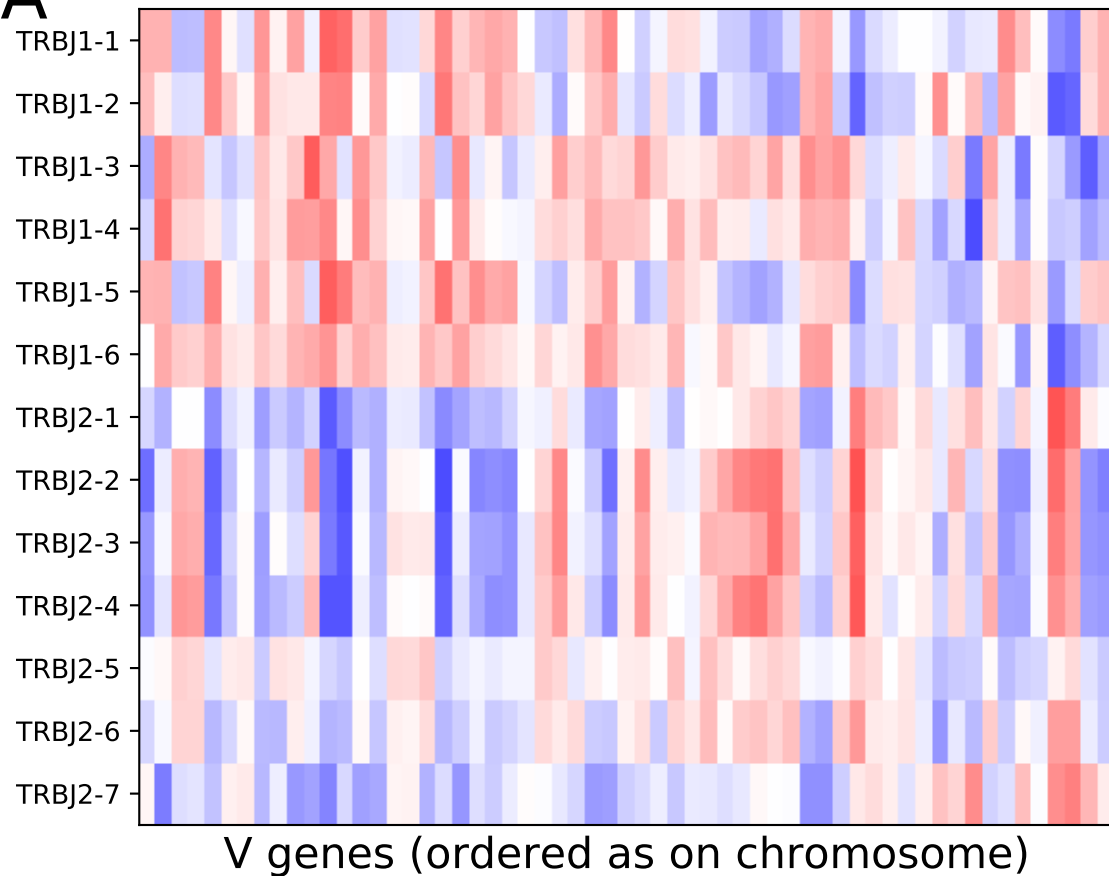**B**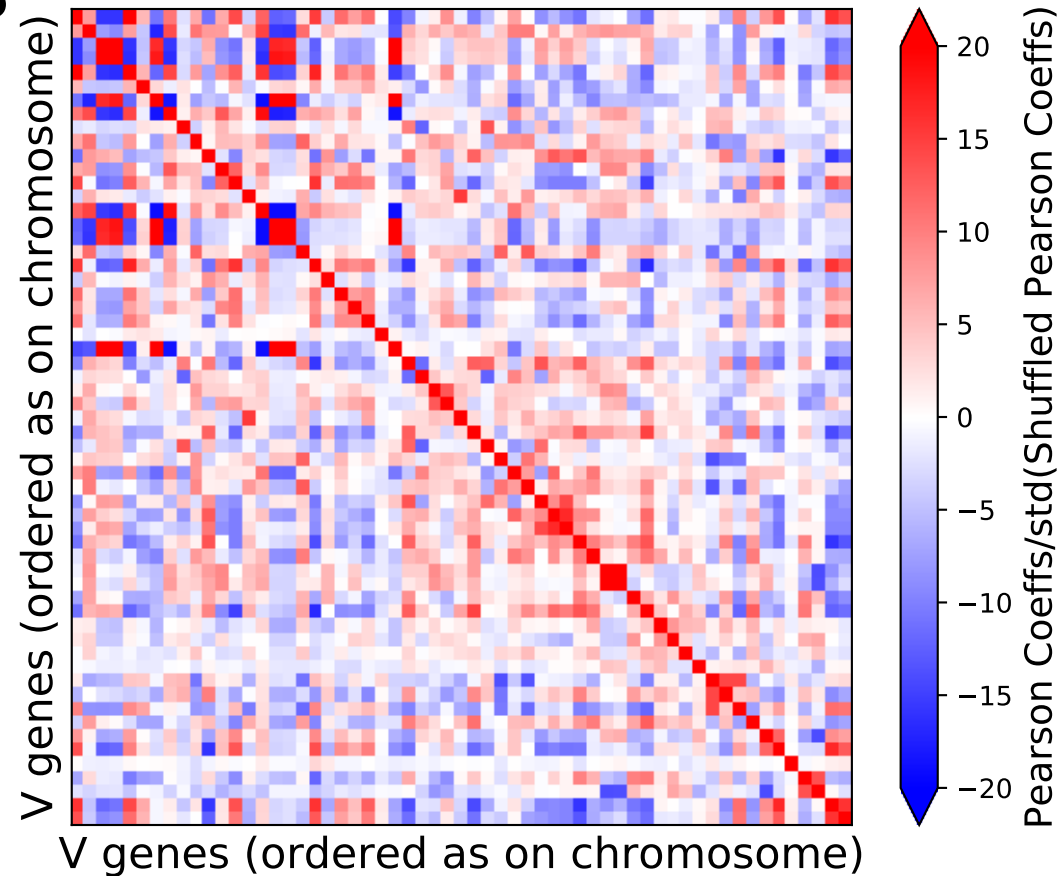

Supplement: S4 Fig — (A) Rescaled Pearson coefficients for V-J correlations across the 651 individual cohort. (B) Rescaled Pearson coefficients for V-V correlations. V genes are ordered by position on the chromosome. While large V-J and V-V correlations exist, no obvious chromosomal structure emerges. Rescaling as in S1 Fig. (PDF) [file pcbi.1008394.s004.pdf]

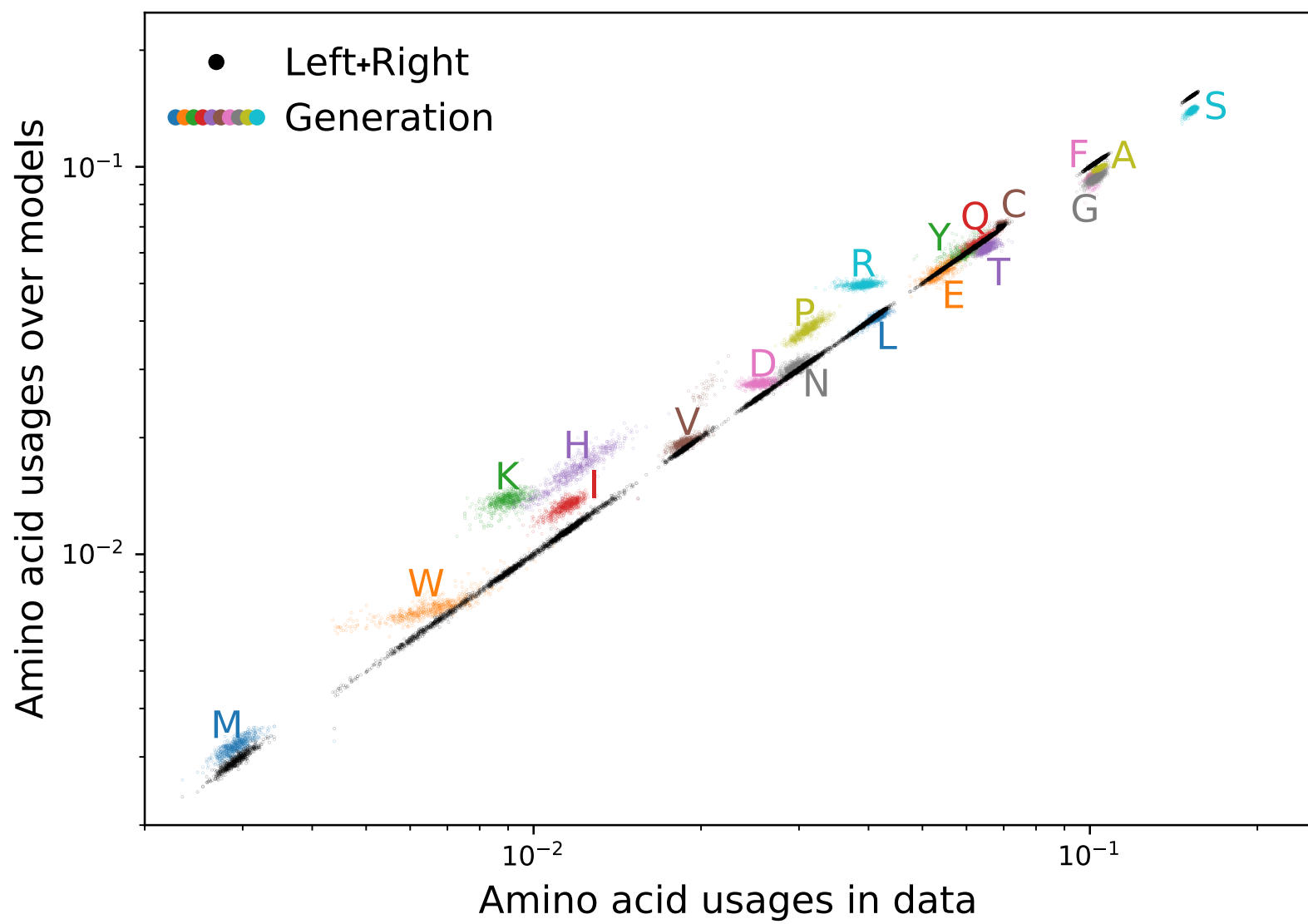

Supplement: S5 Fig — The x-axis is the amino acid usage over the data sequences from a given individual. The y-axis is the amino acid usage over sequences generated from the same individual’s VDJ generation model Pgeni (colored dots, each point is an individual), or the same sequences weighted by the Qi factors from the individual’s Left+Right selection model (black dots). (PDF) [file pcbi.1008394.s005.pdf]

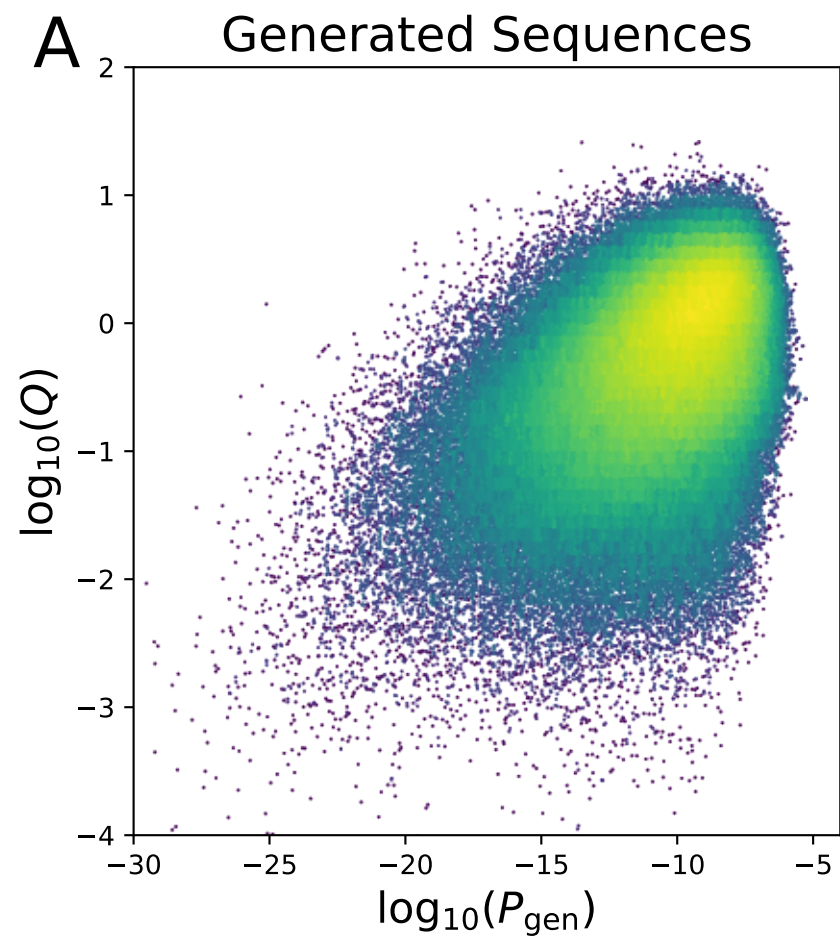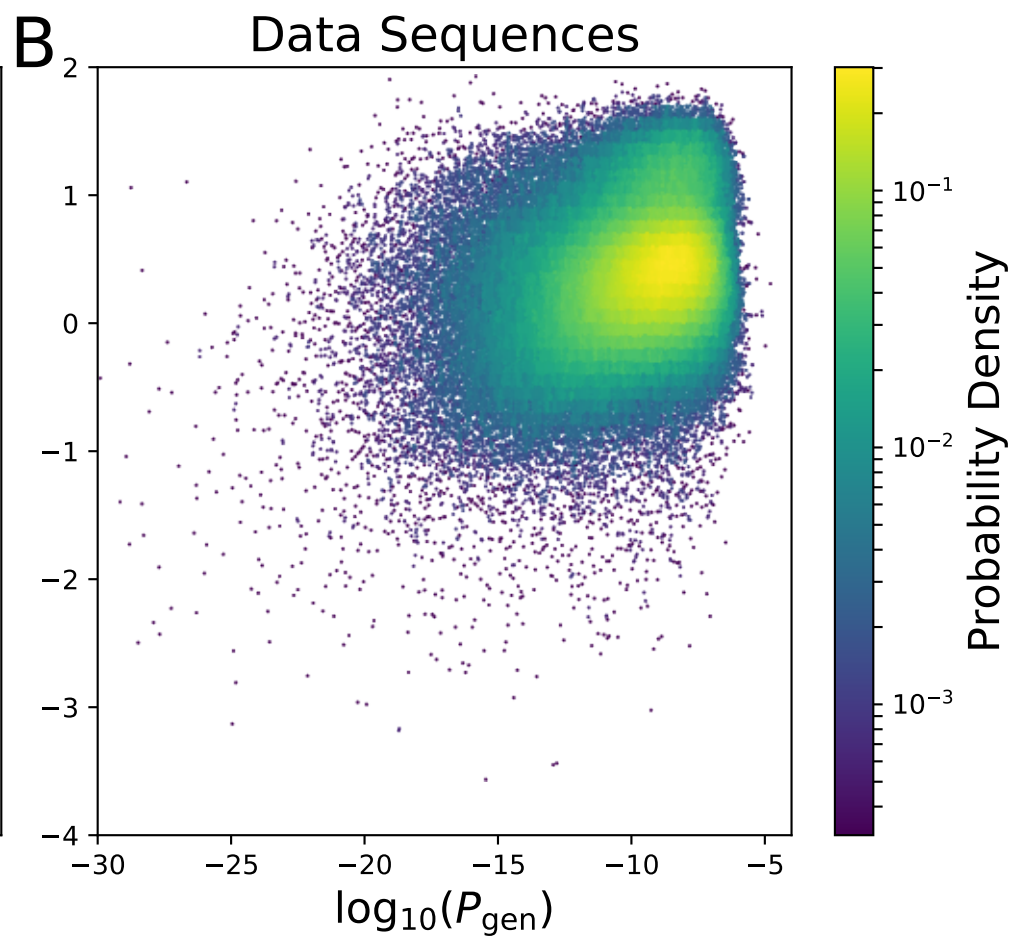

Supplement: S6 Fig — Scatter plots of log10(Quniv) vs log10(Pgenuniv) for (A) generated sequences drawn from Pgenuniv and (B) data sequences used to infer log10(Quniv). The color scale indicates the local probability density of the points (on a log scale). This visualizes the correlation of Pgen and Q as described in Tab. I. Quniv and Ppostuniv are ‘universal’ models learned from sequences randomly drawn from all individuals. (PDF) [file pcbi.1008394.s006.pdf]

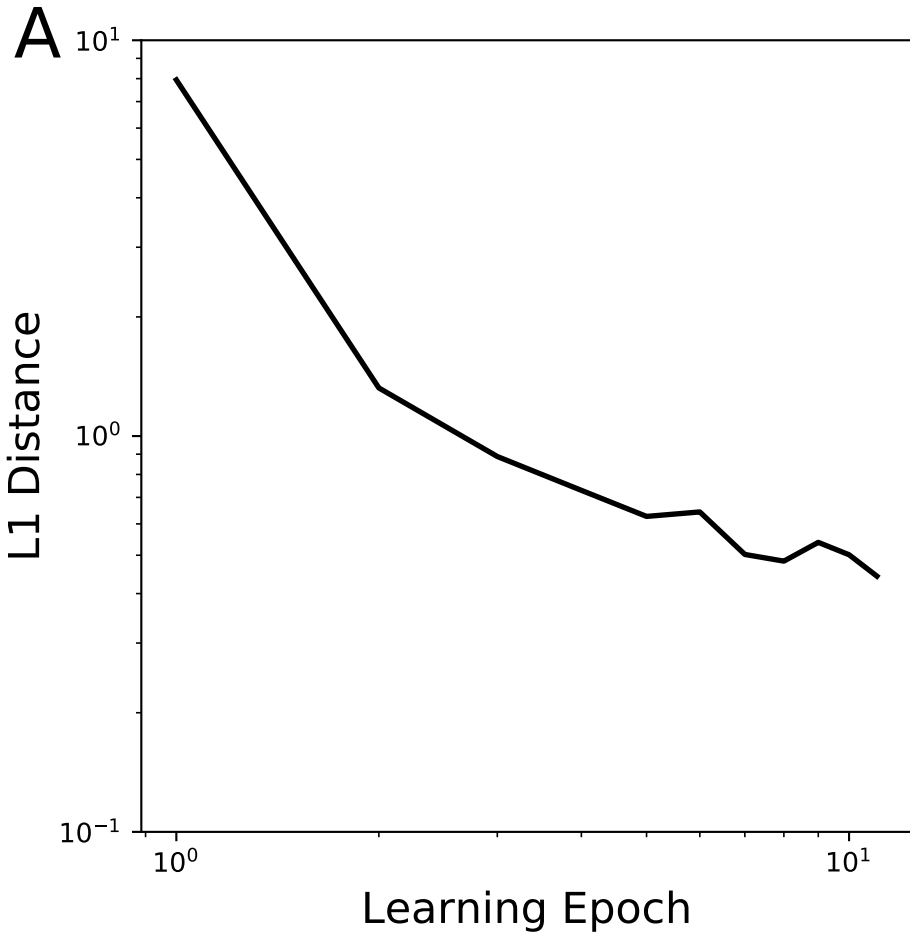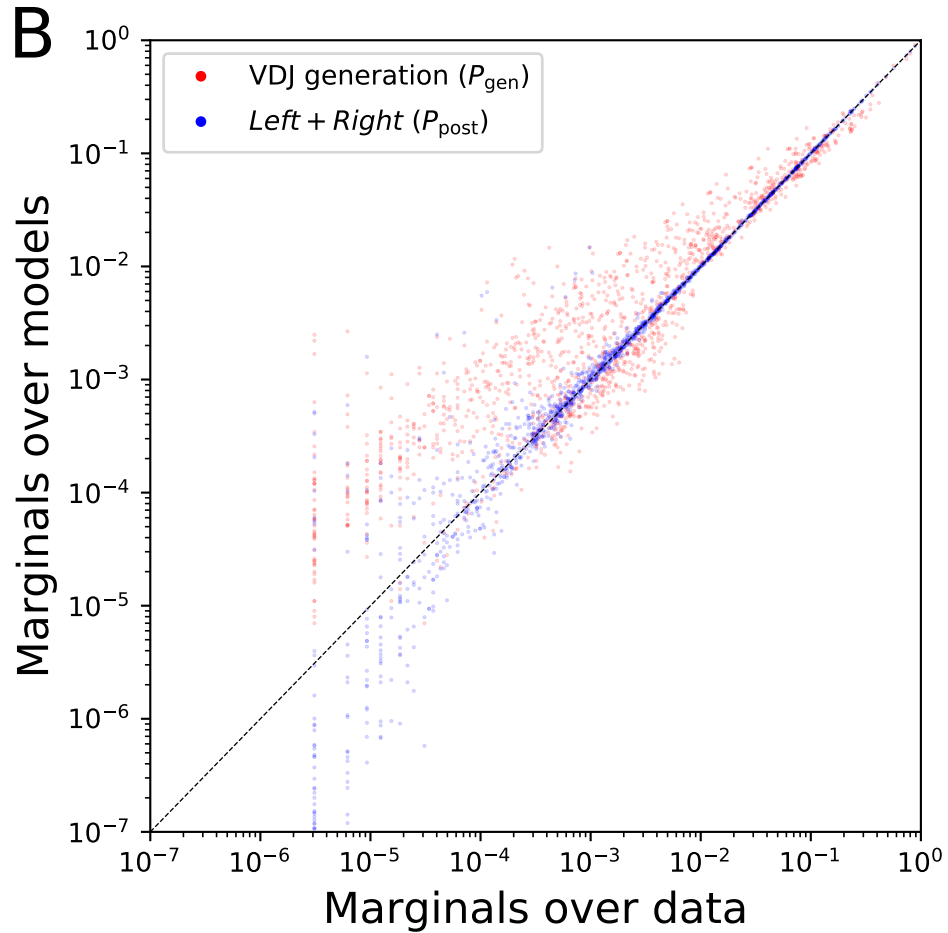

Supplement: S7 Fig — (A) L1 convergence, per learning epoch, of the marginals (or frequencies) between the data features and the model features. (B) Scatter plot of the feature marginals. The x-axis shows the frequencies of features of the data, while the y-axis show the model prediction for the generation model (red) and for Q-weighted Left+Right model (blue). The L1 distance in (A) measures the mean distance between the blue dots and the diagonal. (PDF) [file pcbi.1008394.s007.pdf]

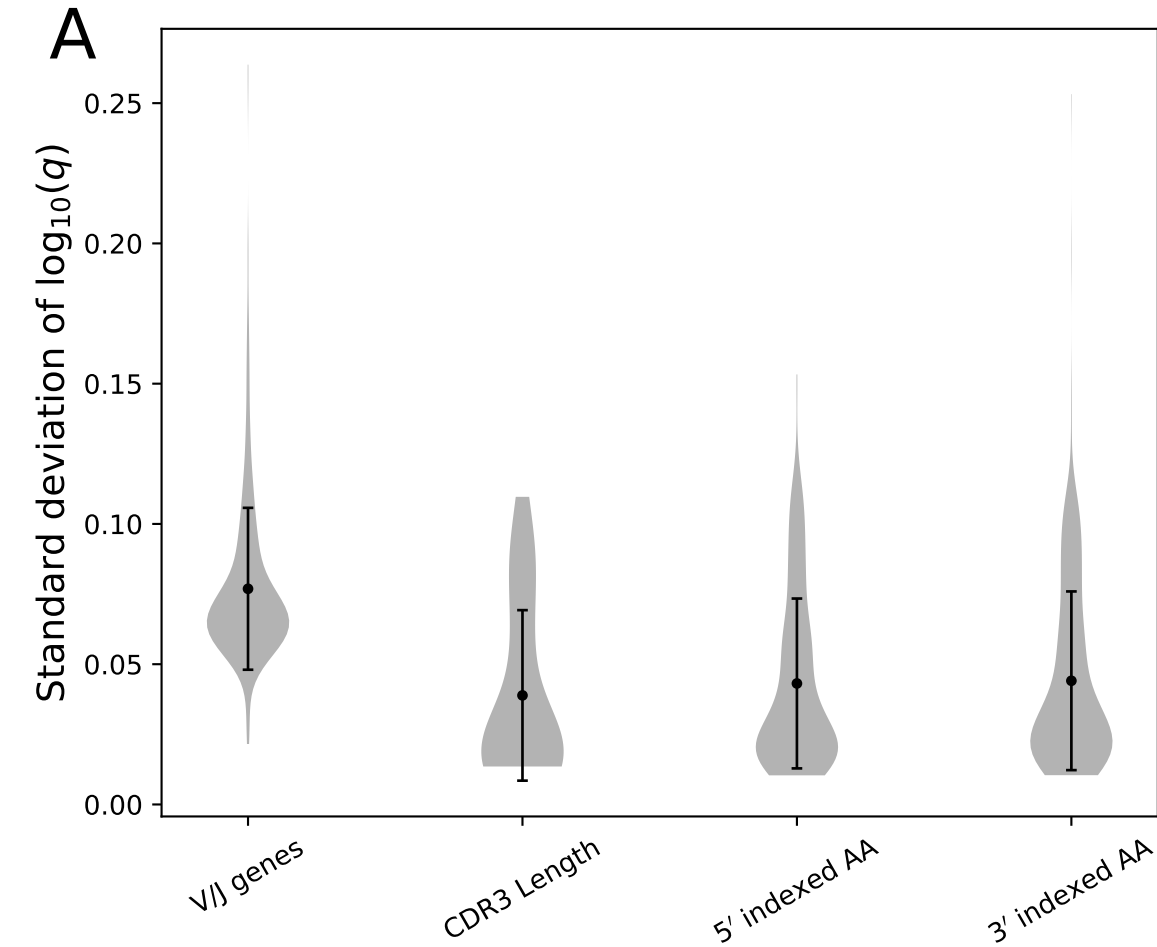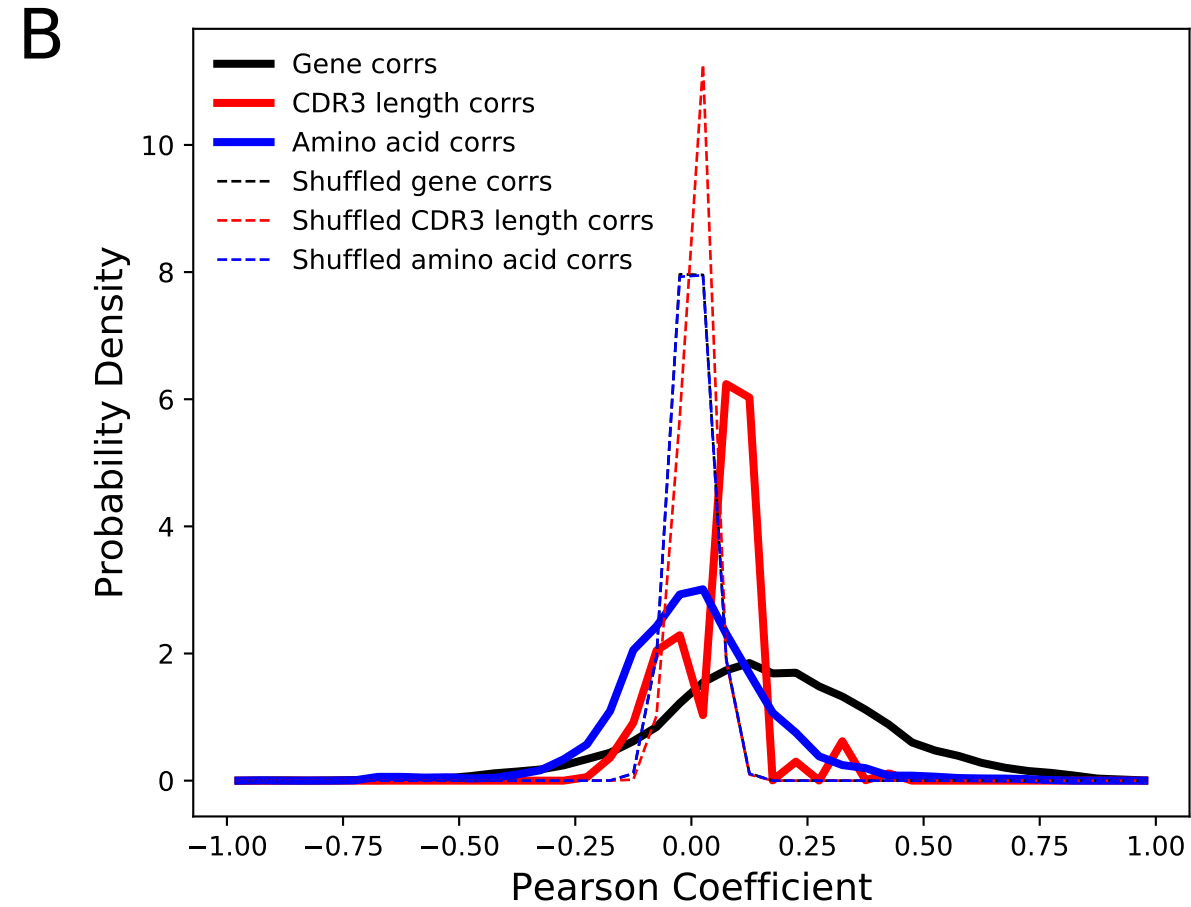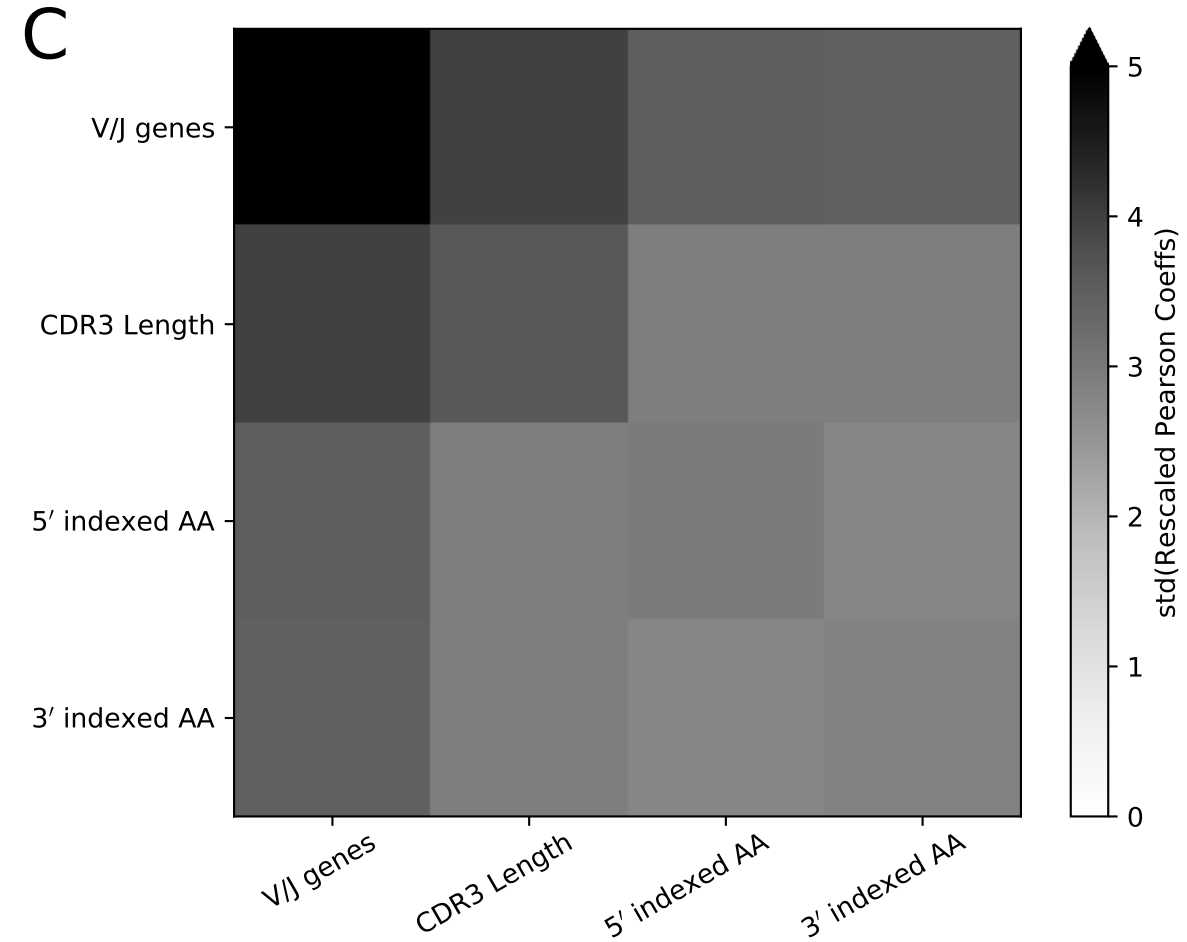

Supplement: S8 Fig — Features are grouped into four types: 1) V/J genes features are the joint V-J features, 2) CDR3 length features, and 3-4) amino acid features indexed either from the conserved cysteine on the 5′ end of the CDR3 or the conserved phenylalanine at the 3′ end. (A) Violin plot of standard deviations of log10(q) for each feature group. (B) Histograms of Pearson correlations within the feature groupings (weighted by feature marginals over the dataset used to infer the universal selection model). (C) Rescaled mean Pearson correlations within and between feature groups (again weighted by feature marginals over the dataset). We note that gene features not only appear more variable than those of other types (A), but are correlated between individuals substantially more than those of other types. (PDF) [file pcbi.1008394.s008.pdf]
